# Cervical cancer survival in a resource-limited setting-North Central Nigeria

**DOI:** 10.1186/s13027-016-0062-0

**Published:** 2016-03-24

**Authors:** Jonah Musa, Joseph Nankat, Chad J. Achenbach, Iornum H. Shambe, Babafemi O. Taiwo, Barnabas Mandong, Patrick H. Daru, Robert L. Murphy, Atiene S. Sagay

**Affiliations:** Department of Obstetrics and Gynecology, University of Jos/Jos University Teaching Hospital, Jos, Plateau State Nigeria; Department of Medicine, Division of Infectious Diseases, Center for Global Health, Feinberg School of Medicine, Northwestern University, Chicago, USA; Department of Pathology, University of Jos/Jos University Teaching Hospital, Jos, Plateau State Nigeria

**Keywords:** Cervical cancer, Anemia, Advanced disease, Survival, North-Central Nigeria

## Abstract

**Background:**

Organized cervical cancer screening services are presently lacking in Nigeria contributing to late presentation and diagnosis of invasive cervical cancer cases (ICCs) at advanced stages in most gynecologic units in Nigeria. We evaluated outcomes of ICCs diagnosed at Jos University Teaching Hospital (JUTH) to better understand factors associated with cervical cancer survival in similar resource limited settings.

**Methods:**

We performed a retrospective cohort study with a prospective follow up data to estimate time from diagnosis to mortality among women diagnosed with ICCs at JUTH. Women who were diagnosed with ICCs between January 2011 and May 2013 were followed up after initial evaluation at JUTH and subsequent referral for specialized treatment in one of the national oncology treatment centers in Nigeria. The main outcome measured was all-cause mortality rate and overall survival (OS) after diagnosis of ICC. The follow up data were updated and observations were censored March 31, 2015. The overall death rate was estimated using the total number of death events and the cumulative follow-up time from diagnosis to death. We conducted Cox proportional hazard regression to assess factors associated with death.

**Results:**

A total of 65 histologically confirmed ICCs were followed up. The median age of the cohort was 50 years with a median parity of 7. The HIV prevalence in the cohort was 8.2 % and the majority (72.3 %) were diagnosed at advanced stages (AD) of ICC. Simple total abdominal hysterectomy (TAH) was performed in 38.9 % of patients who were diagnosed at early stage disease (ED). After a cumulative follow up of 526.17 months, 35 deaths occurred with an overall death rate of 79.8 per 100 women-years. We also found a significantly higher hazard of death in women with AD (HR = 3.3) and baseline anemia (HR = 3.0). In the subgroup of women with ED, the OS was significantly higher for those who had TAH compared to those who did not (26.5 versus 11.6 months respectively).

**Conclusion:**

Advanced stage disease and baseline anemia were independently associated with higher death rate. Cervical cancer patients diagnosed at early stages by non-oncologic specialist in settings lacking the standard of care may benefit from improve survival with simple hysterectomy.

## Background

With over 528,000 new cases of invasive cervical cancer (ICC) and 266,000 deaths reported annually, it is the fourth most common cancer in women worldwide [1, 2]. Cervical cancer screening programs have led to a sustained decline in cervical cancer incidence and mortality in developed countries and prevented premature deaths in underserved populations [3, 4]. This is attributable in part to early detection of invasive cervical cancer, thereby improving survival following treatment [5].

In contrast to the trend in developed countries, Nigeria and other countries in sub-Saharan Africa with limited cervical cancer screening programs are experiencing an upsurge in ICC cases [6]. Indeed, almost 70 % of the global burden of cervical cancer resides in these countries [2]. Cervical cancer screening helps in identifying precancerous conditions of the cervix in asymptomatic women at risk, the treatment of which halts progression to invasive stages. Therefore, Cervical cancer screening does not only lead to significant reduction in incident cases of cancer, but has the additional benefit of providing opportunities for early detection of new cases thereby improving prognosis for survival following treatment. The glooming disparities in cervical cancer incidence and mortality between the developed and developing countries are further evident in the recent global cancer statistics reporting 34.8 new cases and 22.5 deaths from cervical cancer per 100,000 women annually in sub-Saharan Africa [1], compared to North America with a cervical cancer annual incidence of 6.6 per 100,000 women and a mortality of 2.5 per 100,000 women.

The absence of organized cervical cancer screening services in Nigeria, often leads to ICC cases presenting at advanced stages in health facilities lacking infrastructure for effective treatment [7–9]. This situation often limits available care in such hospitals to initial evaluation, symptomatic treatment and referral [7, 9] of confirmed ICCs to the few national treatment centers with chemo-radiation facilities within the country.

In this report, we present descriptive epidemiologic data on cervical cancer patients diagnosed and treated by non-oncologists at a tertiary medical center with limited treatment facilities in Nigeria. Follow up data documenting survival and mortality rates from ICCs are rare in such clinical settings. Therefore, the report is focused on survival of ICC cases and the challenges of managing such cancers in our environment.

## Methods

### Study setting and design

We performed a retrospective cohort study with a prospective follow up data estimating time from diagnosis to death among women diagnosed with ICC in the department of obstetrics and gynecology of Jos University Teaching Hospital (JUTH), north central Nigeria. Women with histologic diagnosis of cervical cancer between January 2011 and May 2013 were included in the study and followed from diagnosis to death, last follow-up or loss to follow up.

### Diagnosis of cervical cancer and initial assessments

Diagnostic evaluation of suspected cases of cervical cancer included examination under anesthesia (EUA) during which clinical staging was made based on the International Federation of Gynecology and Obstetrics pre-2009 revision [10]. Cervical biopsies were obtained for histopathologic confirmation and grading (well-differentiated, moderately differentiated or poorly differentiated). Histologic types were categorized as squamous cell carcinoma, adenocarcinoma or adenosquamous cell carcinoma. Each diagnostic evaluation also included baseline hemogram (Packed Cell Volume), HIV screening (Rapid Determine Test), renal and liver function assessment. Patients also had abdominal and pelvic ultrasound scans, chest X-rays and pre-operative/pre-radiation intravenous urogram (IVU).

### Management of cervical cancer, follow up and main outcome

Throughout the study period, our institution did not have facilities for chemo-radiation and we did not have a trained medical or gynecologic-oncologist in cervical cancer treatment. Due to this limitation, cervical cancer patients received either symptomatic care (including correction of anemia, treatment of infections, and pain control) or referral to Ahmadu Bello University Teaching Hospital (ABUTH), Zaria for chemo-radiation. ABUTH is a tertiary academic medical center equipped with facilities for chemo-radiation located approximately 300 kilometers north-west of Jos. A fraction of the patients who presented in early stages (FIGO stages 1-2A) had simple total abdominal hysterectomy (TAH) performed at the discretion of the attending gynecologist. Simple total abdominal hysterectomy here refers to removal of the uterus with the cervix and upper vaginal cuff without lymph node dissection. All diagnosed ICCs were offered referral to a chemo-radiation treatment facility irrespective of stage at diagnosis or hysterectomy.

Follow up data were obtain from the gynecology outpatient clinic records and via phone calls (patient’s and next-of-kin phone contacts were obtained at diagnosis) to ascertain if chemo-radiation was received after referral to ABUTH. The primary outcome measured was all-cause mortality determined by the occurrence of death following ICC diagnosis within the period of follow up. Time from diagnosis to death (event) or last follow-up, loss to follow up, and March 31, 2015 (censor) was calculated to determine overall mortality rate and survival probabilities for various factors in the cohort.

### Data collection

Demographic, clinical, laboratory, follow up and outcome data were captured in case report forms from the following source documents: individual case files, histopathologic database, nurses’ in-patient notes and hospital discharge summaries. Study variables were entered into an excel spreadsheet and relevant variables were coded for subsequent statistical analyses. The time from diagnosis to mortality was calculated in days and subsequently converted to weeks and lunar months. Patients who did not return to the gynecology clinic and we could not contact on the phone nor ascertain whether they were still alive or had died were considered lost to follow up with observations censored at the last known status date. For this group of patients, we calculated the time from diagnosis to the last date we had information on their survival.

### Statistical analyses

The analytical focus of this study was to determine the all-cause mortality rate and to identify predictive factors that contributes to mortality following diagnosis of cervical cancer given our limited treatment facilities. We generated summary descriptive characteristics using means, medians and proportions where applicable. We compared demographics and clinical characteristics of the patients who were lost to follow up with those who completed follow up with outcome ascertainment. Bivariate statistical analyses of the association between baseline characteristics of the study sample and death were performed using student *t*-test for means of continuous variables and the Pearson chi square or Fisher’s exact tests where applicable for categorical variables. All statistical tests were 2-sided with type 1 error set at 0.05 for statistical significance. To conduct bivariate analyses as well as for the multivariate analyses, cervical cancer FIGO staging was dichotomized to “early” (FIGO Stages IIA or less) and “advanced” (FIGO Stages IIB and greater). Baseline hemogram was similarly dichotomized into baseline anemia (PCV <30 %) and normal hemogram (PCV ≥30 %). Chemo-radiation, TAH, HIV status and mortality were treated as binary variables for determining predictors of death. The overall death rate was estimated using the total number of death events and the cumulative follow-up time from diagnosis to death. We also estimated the rate ratio of death for various factors in the cohort. To estimate the hazard of death following cervical cancer diagnosis, we used time from diagnosis to death in months as a time covariate and death as failure event. Bivariate and multivariate Cox hazard regression modeling were performed to estimate the hazards of death. We also plotted relevant Kaplan-Meier survivor curves to estimate the probability of survival following cervical cancer diagnosis by stage of diagnosis and baseline anemia. We used the Log-Rank test to assess the equality of survival function with *p*-value <0.05 signifying significant difference in survival. Our final predictive model was selected by backward variable selection method. Statistical analyses were performed with STATA version 11.0, College Station, Texas, USA.

#### Ethical consideration and approval

This study was approved by the Institutional Health Research Ethical Committee of the Jos University Teaching Hospital, Jos (*Approval Ref* #: *JUTH*/*DCS*/*ADM*/*127*/*XIX*/*6019*).

## Results

### Baseline demographic and clinical characteristics

**Fig. 1:**
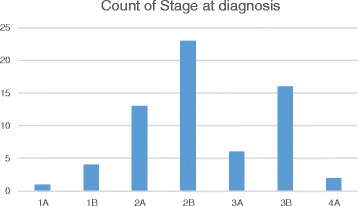
Bar chart of stages of cervical cancer at diagnosis in Jos Nigeria

Between January 2011 and May 2013, 72 cases of suspected cervical cancer were evaluated and managed at JUTH. Sixty-five cases were histologically confirmed and included in this analysis. The median age of the cohort was 50 years with interquartile range (IQR) of 41–60), and median parity of 7 (IQR 5–9). A majority of the patients (72.3 %) were diagnosed at FIGO advanced stages (2B and above) with a median PCV of 28 % (IQR 22.5-35.5). The proportions of the various stages are summarized graphically in Fig. 1 (stage 1a (1.5 %), stage 1b (6.1 %), stage 2a (20.0 %), stage 2b (35.4 %), stage 3a (9.2 %), stage 3b (24.6 %) and stage 4a (3.1)). Of the 65 patients diagnosed, we had HIV status data on 61 and the prevalence was 8.2 %. The histologic types were squamous cell carcinoma (91.0 %), and the predominant histologic grade was moderately differentiated (58.0 %). Other histologic types reported included adeno-squamous cell carcinoma (5.0 %), adeno-carcinoma (2.0 %) and large-cell non-keratinizing squamous cell-carcinoma (2.0 %).

### Clinical intervention after diagnosis

Eighteen (27.7 %) of the 65 cervical cancer patients were diagnosed and categorized as early stage ICC. Of this category, 38.9 % (7/18) had TAH as part of their management at JUTH. There was one case of hysterectomy for advanced disease (FIGO stage 2B) and 1 patient had a laparotomy with a pre-operative staging of FIGO 1B, but tumor was considered inoperable. Sixty-two of the 65 patients offered referral accepted to go to ABUTH for chemo-radiation treatment. Of these 62, 17.7 % (11 out of 62) reported ever receiving any further therapies at the referring hospital (ABUTH). Reported reasons (results not shown) for not receiving chemo-radiation following referral included lack of finances to pay for the service, decision to try traditional medications or seek spiritual care and preference to continue supportive care at JUTH or return to their homes.

### Time to death following cervical cancer diagnosis

Of the 65 that were followed up, 47 (72.3 %) had complete data on time from diagnosis to death and the remaining 18 (27.7 %) had incomplete follow-up data. The total accrued follow-up time from the 65 ICCs in the study was 526.17 months; 35 death events occurred with an all-cause death rate of 79.8 per 100 women-years of follow up after ICC diagnosis. Death rate was significantly higher among women with advanced ICC (rate ratio = 4.7, 95 % CI: 2.0, 12.8, *p*-value 0.001) and those with baseline anemia (rate ratio = 4.8, 95 % CI: 2.3–10.4, *p*-value 0.001). There was significantly lower death rate for those who had chemo-radiation compared with those who did not (rate ratio = 0.33, 95 % CI: 0.11, 0.81, *p*-value 0.007). In the subgroup of women who were diagnosed at early stage ICC, the mean time from diagnosis to death was significantly longer for those who had TAH compare to those who did not (26.5 versus 11.6 months respectively, *p*-value 0.014).

Over two-thirds (68.1 %) of our cohort died after a mean follow up time of 8.3 ± 10.8 months. There was a significantly higher proportion of death in those diagnosed at advanced stages compared to early stages (87.5 % versus 46.7 %, *p*-value 0.009). The mean time to death for advanced disease was 6.9 ± 7.0 months versus 23.7 ± 12.7 months for early disease (*p*-value 0.001). Table 1 summarizes the association between baseline patients’ characteristics and death after diagnosis of ICC.

**Table 1:** Bivariate analyses of the association between patients’ baseline characteristics and mortality from ICCs, Jos Nigeria

Variable	Dead	Alive	*P*-value
Mean Age (yrs)	49.5 ± 12.0	54.4 ± 12.3	0.228*
Mean Parity	6.3 ± 3.1	7.0 ± 2.6	0.458*
Disease stage			
Advanced	28 (87.5)	4 (12.5)	0.009**
Early	7 (46.7)	8 (53.3)	
Chemo-radiation			
Yes	6 (54.5)	5 (45.5)	0.094***
No	28 (80.0)	7 (20.0)	
HIV status			
Positive	4 (80.0)	1 (20.0)	0.743**
Negative	30 (73.2)	11 (26.8)	
Anemia			
Yes	22 (91.7)	2 (8.3)	0.008**
No	13 (56.5)	10 (43.5)	
Follow-up time (Months)	6.9 ± 7.0	23.7 ± 12.7	0.001*

*Student *t*-test, **Fisher exact test, ***Pearson chi square test

### Factors predictive of death

**Fig. 2:**
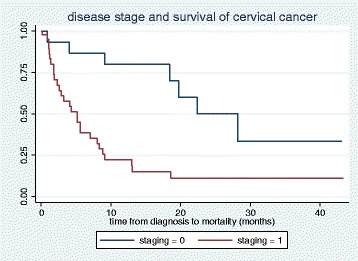
Kaplan Meier graph for survival probabilities of cervical cancer stages at diagnosis in Jos Nigeria. Early cervical cancer is represented with “staging = 0” and Advanced cervical cancer is represented with “staging = 1”. Log-rank *p*-value 0.001

**Fig. 3:**
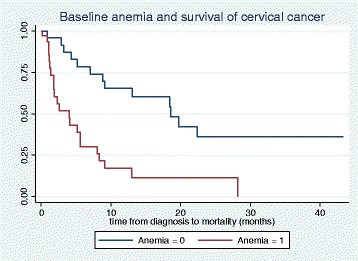
Kaplan Meier graph for survival probabilities of cervical cancer by baseline anemia status in Jos Nigeria. Cervical cancer patients with baseline anemia (PCV <30 %) at diagnosis is represented with “Anemia = 1” and patients who had normal hemogram (PCV ≥30 %) is represented with “Anemia = 0”. Log-Rank *p*-value 0.0002

**Fig. 4:**
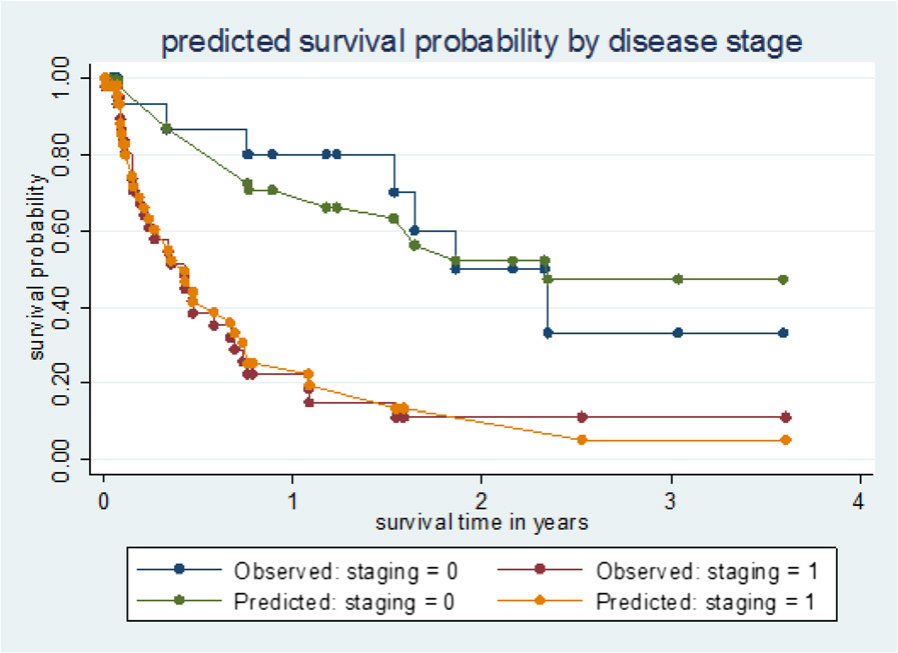
Kaplan Meier graph of observed and predicted survival probabilities for cervical cancer in Jos Nigeria. Early stage cervical cancer is represented with “Staging = 0” and Advanced stage cervical cancer is represented with “staging = 1”

In a Cox regression model, after a total follow up time of 537.1 months, 35 deaths occurred out of the 65 ICCs diagnosed at JUTH. The unadjusted model showed a statistically higher hazard of death in patients with advanced disease (HR = 3.9, 95 % CI: 1.7, 9.2, *p*-value 0.002), and baseline anemia (HR = 3.7, 95 % CI: 1.8, 7.5, *p*-value 0.001). Chemo-radiation showed a trend towards reduced hazard of mortality (HR = 0.45, 95 % CI: 0.19, 1.12, *p*-value 0.088). In the adjusted model including stage at diagnosis, baseline anemia and chemo-radiation; advanced disease and baseline anemia were independently associated with significant hazards of death after diagnosis of ICC (HR = 3.3, 95 % CI: 1.2–8.9, *p*-value 0.021 and HR = 3.0, 95 % CI: 1.4, 6.4, *p*-value 0.006) respectively (Table 2; Figs. 2 and 3). Additionally, Fig. 4 showed the observed and predicted survival probability for early and advanced ICCs. The predicted 2-year survival probability was about 15 % for advanced and 50 % for early disease in our cohort.

**Table 2:** Unadjusted and adjusted Cox regression analyses of factors associated with death after diagnosis of ICCs, Jos Nigeria

Variable	Unadjusted HR (95 % CI)	*p*-value	Adjusted HR (95 % CI)	*p*-value
Advanced stage	3.9 (1.7, 9.2)	0.002	3.3 (1.2, 8.9)	0.021
Baseline anemia	3.7 (1.8, 7.5)	0.001	3.0 (1.4, 6.4)	0.006
Chemo-radiation	0.45 (0.19, 1.12)	0.088	0.9 (0.3, 2.4)	0.815
HIV positive	1.33 (0.46, 3.81)	0.586		

*HR* hazard ratio, *CI* confidence interval

## Discussion

In this retrospective cohort study at a tertiary hospital in Jos Nigeria, a total of 65 cases of cervical cancer were diagnosed over a 28-month period, 72.3 % of which were in advanced stages at presentation. This result reflects the enormous burden of late presentation of ICCs in a tertiary academic medical center with limited oncologic treatment facilities. This finding of late presentation is consistent with a study done in Lagos, Nigeria which found that less than 10 % of cervical cancer patients presented at operable stages with the majority presenting with advanced disease [11]. Similarly, a clinico-pathological analysis of cervical cancer seen in a tertiary health facility in south-eastern Nigeria found that 89.3 % of the patients presented at advanced stages [12]. Other related studies in northern Nigerian settings have documented advanced disease at presentation in 66 %–81 % of cervical cancer patients [13, 14]. These studies highlight the huge challenge posed by late presentation of cervical cancer in a country with very limited treatment facilities and few trained gynecologic oncology specialists. Patients diagnosed at advanced stages have few treatment options and are often limited to either chemo-radiation or palliative care. Authors working in similar settings in Nigeria have documented that even patients diagnosed at early stages often do not have the benefits of surgical intervention partly due to a dearth of skilled gynecologic surgical oncologists [9].

We also found that over two-thirds (68.1 %) of our cohort had died after a mean follow up of 8.3 ± 10.8 months following diagnosis of cervical cancer. A retrospective review of cancer mortality over a 5 year period in a single tertiary institution in Nigeria revealed that cervical cancer was responsible for 44.7 % of all gynecologic cancer mortality during the study period and over 60.6 % of the deaths occurred within 4 weeks of diagnosis [8]. Our study findings showing a high proportion of mortality with a death rate of 79.8 per 100 women-years of follow up further revealed the dismal survival probability for cervical cancer in our institution and in similar settings in Nigeria.

In our predictive model of the factors related to cervical cancer mortality, we found that advanced stage and baseline anemia where significantly associated with hazard of mortality. Mortality in those diagnosed at advanced stages compared to early stages was 87.5 % versus 46.7 % with a mean time to mortality for advanced disease of 6.9 ± 7.0 months versus 23.7 ± 12.7 months for early disease. In the Cox regression model, we found that the predicted 2-year survival probability was about 15 % for advanced disease and 50 % for early disease (Fig. 4). Our predicted survival probability was similar to a Kenyan study which showed a progressively lower survival probability with advancing stage of disease at diagnosis with an overall predicted 2-year survival of less than 20 % [15]. The overall median survival in the Kenyan cohort was 15.0 months following diagnosis and concurrent chemo-radiation therapy at a cervical cancer treatment center.

The mean age at diagnosis of our study sample was 50 years and 27.7 % were diagnosed at early stages out of which 38.9 % had simple hysterectomy prior to referral for chemo-radiation. Although our study was not designed to evaluate the effect of surgery on survival, we observed from our data a potential benefit of simple hysterectomy on survival in patients presenting in our setting in early stages of cancer. The mean survival time for early stage ICCs who had simple hysterectomy was 26.5 months compared to 11.6 months for early stage ICCs who did not have hysterectomy (*P*-value 0.014). Indeed, the role of surgery and the extent to which it should be done for early stage cervical cancer has been a subject of ongoing controversy. There is a growing volume of evidence supporting the practice of minimal surgical resection (i.e. simple hysterectomy) as opposed to radical approaches in early stage disease [16–19]. The proponents of simple hysterectomy point to higher risks of post-surgical morbidity such as lymphedema, urological and sexual dysfunction and other quality of life issues. We acknowledge the limitation of our relatively small sample size in making deductions on the statistical significance of hysterectomy in improving survival for early stage ICCs; however, the observed difference in mean survival time for those who had this intervention may be clinically significant in settings where no standard of care is available for treatment of ICCs. This suggested clinical approach seems sensible in our setting and is supported by our data showing that only 11 (17.7 %) of the 62 patients who accepted referral for chemo-radiation reported receiving this treatment.

The very low completion rate of referrals for chemo-radiation is of concern. Some of the reasons reported for not receiving chemo-radiation were summarized above. They included lack of finances to pay for the service, and decision to pursue traditional medications or spiritual care at home. These highlight some of the worrisome social epidemiologic barriers affecting cervical cancer care in resource limited setting such as Nigeria. Unaffordable cost, for example, is a pervasive factor hindering access to essential health services in Nigeria. The current national health insurance scheme (NHIS) in Nigeria does not provide coverage for cervical cancer prevention or treatment, hence most patients incur heavy out-of-pocket payments [20]. It is likely that high cost of care contributed to late presentation and related high mortality seen in this study. Interventions to surmount this and other barriers to cervical cancer care are needed urgently. We hope to conduct future qualitative research studies to better understand these barriers and how we could overcome them to improve cervical cancer outcome in our population.

A major strength of our study is the documentation of follow-up data from diagnosis to death and we utilized the total accrued follow up time in estimating both the all-cause mortality rate, the hazard of death and the survival probabilities in our cohort. However, we also acknowledge the relatively small cohort size of our study as a limitation to conducting subgroup analysis to rule out potential confounding factors on the effect of surgery, chemo-radiation, HIV status and other factors in predicting cervical cancer survival. We also could not assess progression-free survival (PFS) in our cohort. Even though our study focused primarily on all-cause mortality and survival, we recognize that other outcomes such as PFS and health-related quality of life are important in cervical cancer survivors.

## Conclusions

In conclusion, our study findings show that cervical cancer patients diagnosed at JUTH often have advanced stage disease and very high rates of death. The completion rate of referral for chemo-radiation is unpredictably low and we need to understand and improve the social, physical and economic barriers to accessing such services. Although, it is too early to draw definite conclusions on the effect of simple hysterectomy on survival, our preliminary data suggests a potential benefit in patients diagnosed in early cancer stages particularly in our clinical settings where the optimal standard of care is often not available. Finally, the high mortality rate seen in this cohort, represents a clear need for setting up organized cervical cancer screening which offers opportunities for detection and treatment of precancerous cervical lesions thereby halting progression to invasive cervical cancer stages. We also believe that such organized screening program will provide additional benefits in early detection of cervical cancer cases which could be treated with improved prognosis for survival.
